# Effects of Benzo[a]pyrene on Targeted Therapy Response and Platelet-Activating Factor-Receptor-Mediated Microvesicle Particle Release in Non-Small Cell Lung Cancer

**DOI:** 10.3390/medsci14020301

**Published:** 2026-06-11

**Authors:** Riya Rawal, Anita Thyagarajan, Ravi P. Sahu

**Affiliations:** Department of Pharmacology and Toxicology, Boonshoft School of Medicine, Dayton, OH 45435, USA; rawal.9@wright.edu

**Keywords:** non-small cell lung cancer, benzo[a]pyrene, platelet-activating factor-receptor, epidermal growth factor receptor, targeted therapy, microvesicle particles

## Abstract

Background/Objectives: Non–small cell lung cancer (NSCLC) is a leading cause of cancer-related mortality, driven by invasive behavior and frequent resistance to systemic therapies. Epidermal growth factor receptor tyrosine kinase inhibitors (EGFR-TKIs) benefit patients with EGFR-mutant NSCLC, but their efficacy is often limited by tumor-intrinsic and environmental resistance mechanisms. Benzo[a]pyrene (BaP), a ubiquitous polycyclic aromatic hydrocarbon from tobacco smoke, combustion, and dietary sources, is a known carcinogen; however, its role in modulating therapeutic responses is poorly understood. Studies, including ours, implicate the platelet-activating factor-receptor (PAFR) pathway in mediating environmental pollutant and therapy-induced effects on tumor growth and microvesicle particle (MVP) release. We hypothesized that PAFR activation mediates BaP-induced NSCLC progression and influences EGFR-TKI responses. Methods: We assessed the effects of BaP, PAFR agonist CPAF, EGFR-TKIs, and their combinations on cell viability, proliferation, migration, anchorage-independent growth, and MVP secretion. Results: BaP did not alter cell survival but significantly increased migration, growth, colony formation, and MVP release, similar to CPAF, and these effects were blocked by a PAFR antagonist or acid sphingomyelinase inhibitor. Notably, BaP did not significantly reduce EGFR-TKI efficacy at tested concentrations. Conclusions: These results show that environmental carcinogens modulate NSCLC behavior through PAFR signaling without compromising EGFR-TKI responsiveness, highlighting PAFR as a potential therapeutic target.

## 1. Introduction

Lung cancer is one of the most common malignancies and remains the leading cause of cancer-related mortality worldwide [1,2]. Despite significant advances in cancer treatment strategies, including chemotherapy, radiotherapy and immunotherapy, chemoresistance and metastasis continue to pose major challenges to patient recovery [3,4]. Non-small cell lung cancer (NSCLC) accounts for the majority of lung cancer cases, and many patients harbor activating mutations in oncogenes, such as the epidermal growth factor receptor (EGFR) [5,6]. Consequently, targeted therapy has become a standard-of-care approach, with tyrosine kinase inhibitors (TKIs) representing one of the most effective treatments [5,6]. Nevertheless, various factors, including environmental pollution, may influence the therapeutic response to EGFR-TKIs [7].

Benzo[a]pyrene (BaP), a polycyclic aromatic hydrocarbon, is recognized as one of the major environmental carcinogens [6,8]. It is primarily generated through tobacco smoking, vehicle exhaust, and incomplete combustion of organic materials [6]. Numerous studies have demonstrated that BaP plays an important role in modulating the growth of various cancers, including lung cancer [9]. Mechanistically, BaP is a well-established ligand of the aryl hydrocarbon receptor (AhR), through which it mediates xenobiotic stress responses and carcinogenic signaling in lung epithelial cells [10]. In addition, emerging evidence suggests that BaP exposure may influence extracellular vesicle (exosome/microvesicle) biogenesis and alter vesicle-associated microRNA cargo in both in vitro and in vivo models, indicating a potential role for EV-mediated intercellular communication in environmental toxicant-induced biological responses [11]. Although several signaling pathways have been shown to mediate BaP-induced lung cancer progression [12,13,14], the involvement of a potent phospholipid-sensitive G-protein coupled, platelet-activating factor-receptor (PAFR) pathway in BaP-induced effects has not been studied.

Recent studies have shown that PAFR is expressed in NSCLC cell lines, and its activation induces enhanced cell invasion and proliferation [14,15]. Notably, PAFR activation also stimulates the release of large extracellular vesicles, known as microvesicle particles (MVPs), in response to diverse reactive oxygen species (ROS)-generating stimuli [15]. Given that BaP induces ROS production and proliferation of lung cancer cells, we hypothesized that BaP may stimulate MVP release through activation of the PAFR pathway [16,17]. In lung cancer, MVPs have been shown to play a critical role in modulating therapeutic responses via the activation of PAFR signaling [17,18]. In addition, targeted therapies such as EGFR-TKIs have also been reported to stimulate MVP release [17]. However, the effect of BaP exposure on the efficacy of EGFR-TKIs remains incompletely understood, and whether BaP induces MVP release has not yet been investigated. Therefore, the present study aimed to determine the effects of BaP on EGFR-TKI efficacy and PAFR-mediated MVP release in NSCLC.

## 2. Materials and Methods

### 2.1. Reagents

F-12K (CAS No. SH30526.01) and RPMI-1640 (CAS No. SH30527.02) culture media and Hank’s Balanced Salt Solution, also referred to as HBSS (CAS No. SH30268.02), were obtained from Cytiva HyClone Laboratories (Logan, UT, USA). Benzo[a]pyrene (CAS No. B1760), desipramine (CAS No. 58-28-6), imipramine (CAS No. 113-52-0), dimethyl sulfox-ide (DMSO, CAS No. 67-68-5), sulforhodamine B (CAS No. 3520-42-1), and crystal violet (CAS No. 548-62-9) were purchased from Sigma-Aldrich (St. Louis, MO, USA). The PAFR agonist (CPAF, CAS No. 91575-58-5), PAFR antagonist (WEB2086, CAS No. 105219-56-5), gefitinib (CAS No. 184475-35-2), and erlotinib (CAS No. 183321-74-6) were obtained from Cayman Chemical Co. (Ann Arbor, MI, USA). Trichloroacetic acid (TCA, CAS No. 76-03-9) and ethanol (CAS No. 64-17-5) were purchased from Fisher Scientific (Pittsburgh, PA, USA). Agarose (CAS No. 15510-019) was purchased from Invitrogen (Carlsbad, CA, USA), Giemsa stain (CAS No. 51811-82-6) was purchased from Fluka (Ronkonkoma, NY, USA), and methanol (CAS No. 67-56-1) was purchased from Maxtite (Decatur, AL, USA).

### 2.2. Cell Culture

Human NSCLC cell lines, A549 and H1299, used in this study were a kind gift from Dr. Weiwen Long (Wright State University). These cell lines are available in American Type Culture Collection (ATCC). A549 cells (CCL-185^TM^) were isolated from the lung tissue of a White, 58-year-old male with lung adenocarcinoma, which harbor a KRAS (G12S) mutation while expressing wild-type TP53 [19,20]. H1299 cells (also referred to as NCI-H1299; CRL-5803^TM^) were derived from the lung of a White, 43-year-old, male patient with large cell carcinoma. This cell line harbors null TP53 [19,21]. These cell lines were selected as representative NSCLC models expressing functional PAFR, as supported by our previous studies [15]. A549 cells were cultured in F-12K medium, supplemented with 10% fetal bovine serum (FBS), 1% of penicillin/streptomycin, 1% of L-glutamine, containing glucose and sodium pyruvate. H1299 cells were grown in RPMI-1640 medium supplemented with the same concentrations of FBS, penicillin/streptomycin, L-glutamine, glucose and sodium pyruvate. All cell cultures were incubated at 37 °C in a humidified atmosphere containing 5% CO_2_ and 95% relative humidity.

### 2.3. Cell Survival Assay

Cell survival was evaluated using the sulforhodamine B (SRB) assay. Following the indicated treatments, cells were fixed with 10% (*w*/*v*) trichloroacetic acid for 1 h at room temperature, washed with double-distilled water, and stained with 0.4% (*w*/*v*) SRB prepared in 1% (*v*/*v*) glacial acetic acid. Excess dye was removed by washing with 1% acetic acid, and plates were air-dried. The protein-bound SRB was solubilized in 10 mM Tris [tris(hydroxymethyl)aminomethane], and absorbance was measured at 570 nm using a BioTek Cytation 5 or BioTek Synergy H1 microplate reader (Agilent Technologies, Inc., Santa Clara, CA, USA). Cell viability was calculated by normalizing absorbance values to the untreated control group.

### 2.4. Cell Proliferation Assay

Cell proliferation was quantified using the crystal violet assay. After the indicated treatments, cells were washed with cold PBS, fixed with ice-cold methanol, and stained with 0.4% (*w*/*v*) crystal violet for 5–10 min at room temperature. Excess stain was removed by washing three times with double-distilled water, and the plates were air-dried. The cell-associated dye was solubilized in 1% sodium dodecyl sulfate (SDS), and absorbance was measured at 540 nm using a BioTek Synergy H1 microplate reader (Agilent Technologies, Inc., Santa Clara, CA, USA). Data were normalized to the untreated control group.

### 2.5. Cell Migration Assay

Cell migration was evaluated using a wound healing assay. A549 cells were seeded in 12-well plates and allowed to reach 80–90% confluency. A linear scratch was generated using a sterile 1 mL pipette tip, and detached cells were removed by washing with PBS. Cells were then subjected to the indicated treatments, and wound closure was imaged using a Leica DMi1 microscope (Leica Microsystems, Wetzlar, Germany) at 0, 24, and 48 h. Migration was quantified using ImageJ version 1.54g (Washington, DC, USA) software and expressed as the percentage of wound closure relative to the initial scratch width.

### 2.6. Colony Formation Assay

Anchorage-independent growth was assessed using a soft agar colony formation assay in 24-well plates. A base layer of 0.6% agarose prepared in F-12K medium was added to each well and allowed to solidify. A549 cells were seeded at a density of 6000 cells per well, resuspended in culture medium, subjected to the indicated treatments, and mixed with agarose to a final concentration of 0.3%. This cell-agarose suspension was overlaid onto the base layer and allowed to solidify, followed by the addition of complete medium as a top layer. Fresh medium was replenished every 2 days, and plates were incubated for 2 weeks. Colonies were then washed, fixed with methanol, air-dried, stained with Giemsa stain (1:50 dilution), and imaged using a Biotek Cytation5 Imaging Reader microscope (Agilent Technologies, Inc., Santa Clara, CA, USA).

### 2.7. Microvesicle Particle Isolation and Analysis

The vesicles analyzed in this study were microvesicle particles (MVPs), isolated using our previously established protocol and operationally defined based on their size range (100–1000 nm), thereby distinguishing them from other subcellular vesicles, such as smaller exosomes and larger apoptotic bodies. For this, cells were cultured to 80–90% confluency and washed three times with phenol red-free Hank’s balanced salt solution (HBSS). Cells were then incubated in HBSS supplemented with 1% bovine serum albumin (BSA) along with the indicated treatments for 4 h. Following incubation, culture supernatants were collected and centrifuged at 2000× *g* for 20 min to remove cellular debris, followed by ultracentrifugation of the supernatants at 20,000× *g* for 70 min to pellet microvesicle particles (MVP). The isolated MVP pellets were resuspended in 100 µL PBS, and particle concentration and size distribution were determined using a NanoSight NS300 instrument (NanoSight Ltd.; Malvern Panalytical, Worcestershire, UK).

### 2.8. Statistical Analysis

The statistical analysis was assessed by GraphPad Prism Software version 7.05 (GraphPad software, San Diego, CA, USA). All the in vitro experiments were repeated at least three times. Data were analyzed by *t*-tests, one-way ANOVA, and two-way ANOVA with post hoc Tukey’s multiple comparison tests. The ImageJ software was used to quantify cell migration images.

## 3. Results

### 3.1. Effect of BaP on Cell Survival in NSCLC

**Figure 1:**
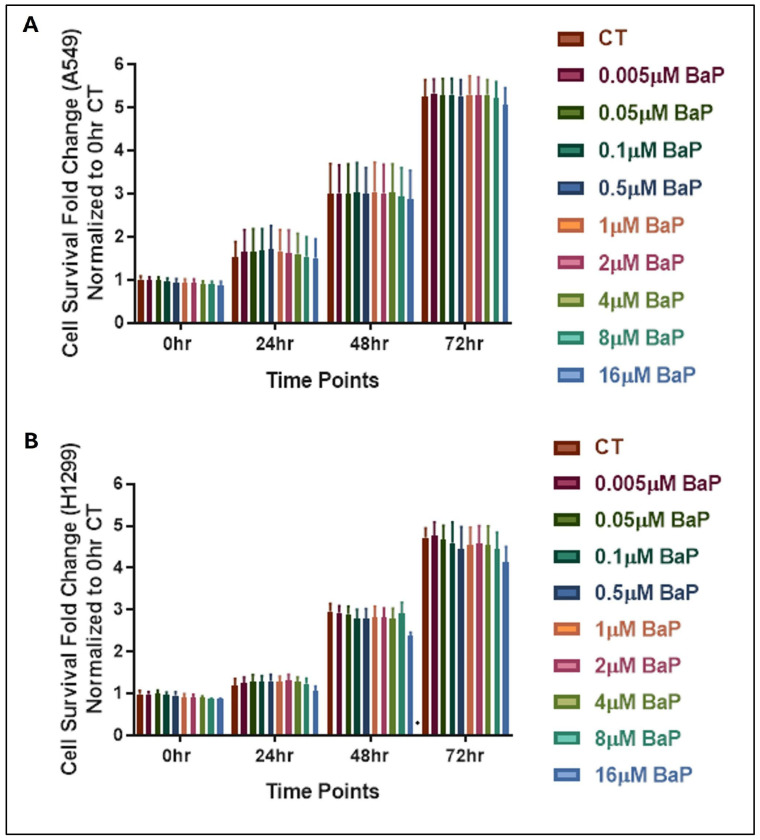
(**A**,**B**). Effect of BaP on cell survival of lung cancer cells. (**A**) A549 and (**B**) H1299 cells (2500 cells/well) were seeded in 96-well plates and incubated overnight. Cells were treated with vehicle control (0.1% ethanol) or increasing concentrations of benzo[a]pyrene (BaP; 0.005–16 µM) and incubated for 0, 24, 48, and 72 h. Data were normalized to the 0 h vehicle control and expressed as percent cell survival relative to the control. Data are mean ± SEM of at least 3 independent experiments.

Our first study examined the dose- and time-dependent effect of BaP on cell survival. For this, A549 and H1299 NSCLC cell lines were treated with BaP at concentrations ranging from 0.005 µM to 16 µM. Cell survival was assessed by the SRB assay after 24, 48, and 72 h of incubation. To determine the baseline effects, cells were stained immediately following BaP treatment (0 h). The results showed that BaP did not significantly affect the cell viability in either cell line at 0, 24, 48, and 72 h compared with their respective controls (Figure 1A,B). However, at the highest concentration tested (16 µM), BaP caused a modest non-significant reduction in H1299 cell viability at the 48 and 72 h time points (Figure 1B). These findings suggest that BaP at concentrations ≤ 8 µM does not significantly influence the survival of these lung cancer cells.

### 3.2. Effect of BaP on the Efficacy of EGFR-TKIs via Cell Migration in NSCLC Models

**Figure 2:**
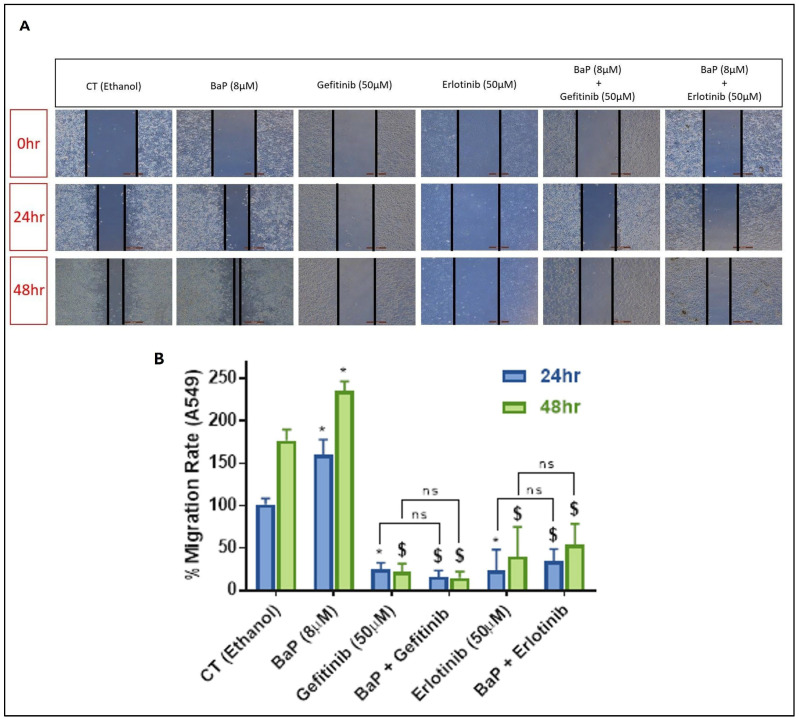
Effect of BaP and EGFR-TKIs on A549 cell migration. A549 cells were seeded at a density of 0.2 × 10^6^ cells/well in 12-well plates. Following overnight incubation, a linear scratch was generated using a sterile 1 mL pipette tip, and detached cells were removed by PBS washes. Cells were subsequently treated with Control (0.1% Ethanol), BaP (8 µM), Gefitinib or Erlotinib (50 µM), and their combinations. For combination treatments, cells were pre-incubated with BaP for 1 h, followed by EGFR-TKIs. (**A**) Representative phase-contrast images of wound closure at 24 and 48 h taken from a Leica DMi1 microscope are shown. (**B**) Quantitative analysis of % migration rate is presented at 24 and 48 h. Data are mean ± SEM of at least 3 independent experiments. The signs * (*p* < 0.05) and $ (*p* < 0.0001) denote statistical significance differences between CT and BaP or Gefitinib or Erlotinib at 24 and 48 h time points. The sign $ (*p* < 0.0001) denotes statistical significance difference between BaP vs. BaP + Gefitinib and BaP vs. BaP + Erlotinib at 24 and 48 h time points. The sign “ns” denotes a non-significant difference between Gefitinib vs. BaP + Gefitinib and Erlotinib vs. BaP + Erlotinib at 24 and 48 h time points. The scale bar for panel (**A**) is 200 µm.

To evaluate the effects of BaP alone and in combination with EGFR-TKIs on the migratory ability of lung cancer cells, a wound scratch assay was performed. In initial experiments, comparable survival responses to BaP were observed in both A549 and H1299 cell lines; therefore, A549 cells were selected as a representative model for subsequent migration assays. As BaP did not affect cell survival at concentrations up to 8 µM (Figure 1A,B), this concentration was selected to assess its impact on cell migration. Similarly, we chose IC_50_ concentrations of the EGFR-TKIs (Gefitinib and Erlotinib) based on our previous report using the same cell lines [22]. Cells were treated with or without BaP and these therapeutic agents, and cell migration was analyzed and quantified 24 and 48 h post-treatment. As shown in Figure 2A (representative images) and Figure 2B (quantification), BaP significantly increased cell migration compared with the respective controls at both time points. In contrast, treatment with EGFR-TKIs significantly inhibited wound closure. However, when cells were treated with BaP in combination with gefitinib or erlotinib, wound closure remained significantly inhibited compared with EGFR-TKIs alone. These findings indicate that EGFR-TKIs effectively suppress cell migration and that exposure to BaP does not compromise their inhibitory efficacy at either time point.

### 3.3. Effects of BaP and PAFR Activation on the Efficacy of EGFR-TKIs via Cell Proliferation in NSCLC Models

**Figure 3:**
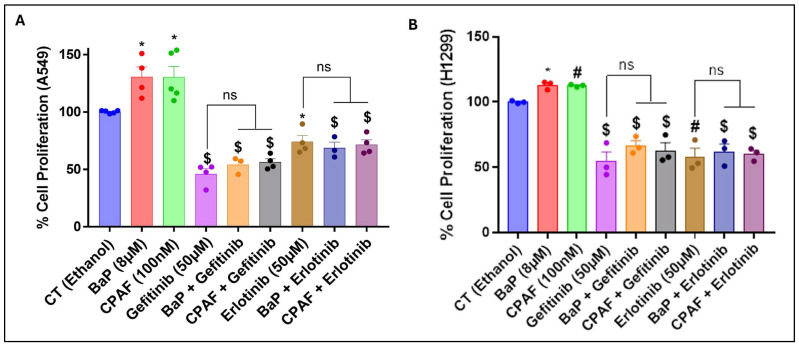
Effects of BaP and CPAF in combination with EGFR inhibitors on proliferation of (**A**) A549 and (**B**) H1299 cells. A549 and H1299 cells (2500 cells/well) were seeded in 96-well plates and treated with vehicle control (0.1% Ethanol), BaP (8 µM), CPAF (100 nM), Gefitinib or Erlotinib (50 µM), or their respective combinations as indicated. Cell proliferation was measured by the crystal violet assay, and absorbance was measured at 540 nm using a Biotek Synergy H1 Microplate Reader. Data are mean ± SEM of at least 3 independent experiments, normalized to the vehicle control, and expressed as % Cell proliferation relative to control. The signs * (*p* < 0.05), # (*p* < 0.001) or $ (*p* < 0.0001) denote statistical significance difference between control and individual treatments. The sign $ (*p* < 0.0001) denotes statistical significance difference between individual treatment (BaP or CPAF) and their respective combination treatments with Gefitinib or Erlotinib, and “ns” indicates non-significance between Gefitinib vs. BaP + Gefitinib, Gefitinib vs. CPAF + Gefitinib and also Erlotinib vs. BaP + Erlotinib, Erlotinib vs. CPAF + Erlotinib.

To validate the effects of BaP on the efficacy of EGFR-TKIs (Figure 2) and to assess the role of PAFR activation in this context, we evaluated the proliferative response of lung cancer cells. Specifically, we examined whether the PAFR agonist CPAF modulates the activity of Gefitinib and Erlotinib. A549 and H1299 cell lines were treated with or without BaP, CPAF, Gefitinib or Erlotinib alone, or BaP or CPAF combination with Gefitinib and Erlotinib, and cell proliferation was measured 48 h post-treatment. As shown in Figure 3A,B, BaP and CPAF alone significantly enhanced cell proliferation, whereas EGFR-TKIs markedly inhibited proliferation. Notably, co-treatment with BaP or CPAF did not significantly alter the antiproliferative effects of Gefitinib or Erlotinib when used at IC_50_ concentration (Figure 3A,B) or IC_25_ concentration in either cell line. These findings indicate that BaP and CPAF do not compromise the efficacy of EGFR-TKIs at the tested concentrations in these lung cancer cell models.

### 3.4. Effects of BaP and PAFR Activation on the Efficacy of EGFR-TKIs via Anchorage Independent Colony Formation in NSCLC Models

**Figure 4:**
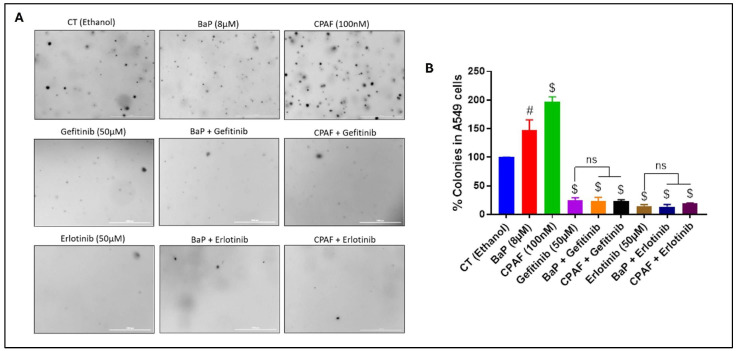
Effects of BaP and CPAF in colony formation in NSCLC: (**A**) A549 cells were seeded (6000/well) in 24-well plates. The cells were treated with BaP (8 µM), CPAF (100 nM), Gefitinib or Erlotinib (50 µM), and their above indicated combinations. After two weeks, the colonies were stained and imaged using a Biotek Cytation5 Imaging Reader microscope. (**B**) The data are presented as mean ± SEM of at least 3 independent experiments and presented as percentages of colonies relative to the control. The signs **#** (*p* < 0.001) or $ (*p* < 0.0001) denote statistically significant difference between control and individual treatments. The sign $ (*p* < 0.0001) indicates comparison of BaP with both BaP + Gefitinib and BaP + Erlotinib, as well as CPAF with both CPAF + Gefitinib and CPAF + Erlotinib combinations However, sign “ns” indicates non-significant difference between Gefitinib with BaP + Gefitinib and CPAF + Gefitinib and Erlotinib with BaP + Erlotinib and CPAF + Erlotinib. The scale bar for panel (**A**) is 1000 µm.

To further assess the effects of BaP and CPAF on the efficacy of EGFR-TKIs, an anchorage-independent clonogenic assay was performed. Given that similar proliferative responses to BaP, CPAF, and EGFR-TKIs were observed in both A549 and H1299 cell lines (Figure 3), A549 cells were subsequently selected as a representative model for this assay and treated with or without BaP, CPAF, EGFR-TKIs alone, as well as in combination. Colony formation was evaluated by Giemsa staining. As shown in Figure 4A (representative pictures) and Figure 4B (quantification), BaP and CPAF alone significantly increased colony formation compared to the control. In contrast, treatment with EGFR-TKIs markedly reduced colony formation, and this inhibitory effect was maintained in the presence of BaP or CPAF. These results confirm that neither BaP nor CPAF significantly alter the antiproliferative efficacy of EGFR-TKIs in this NSCLC model under the experimental conditions tested.

### 3.5. Effect of BaP on PAFR-Mediated MVP Release

**Figure 5:**
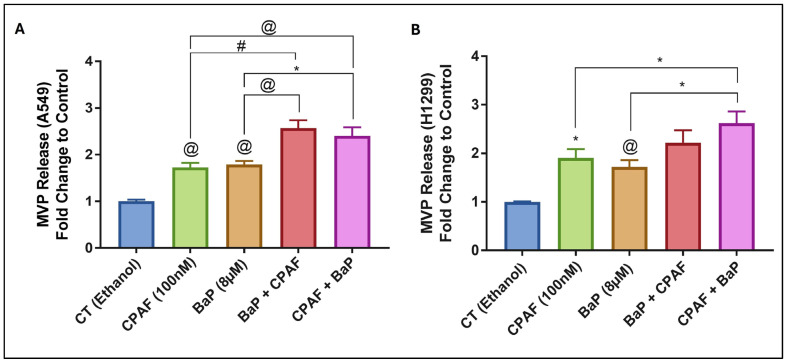
Effects of BaP and CPAF alone or their combination on MVP release in NSCLC: (**A**,**B**) A549 and H1299 cells were plated in 6-well plates, grown to 80–90% confluency, and treated with BaP (8 µM), CPAF (100 nM), or the BaP + CPAF or CPAF + BaP combination. After 4 h, supernatants were collected and subjected to differential centrifugations. The pelleted MVPs were resuspended in 100 µL sterile PBS and quantified by NanoSight tracking analysis. Data are mean ± SEM of at least 3 independent experiments and represented as MVP release fold change to control. The signs * (*p* < 0.05) or @ (*p* < 0.01) indicate statistically significant differences between control and BaP or CPAF alone treatments. However, signs @ (*p* < 0.01) and # (*p* < 0.0001) denote statistically significant difference between CPAF with BaP + CPAF or CPAF + BaP, and * (*p* < 0.05) or @ (*p* < 0.01) indicate differences between BaP with BaP + CPAF or CPAF + BaP.

To determine the effect of BaP on MVP release as well as to assess whether it modulates PAFR-mediated MVP release, H1299 and A549 cell lines were treated with or without BaP and CPAF alone or their combinations. MVPs were subsequently isolated and quantified by NanoSight tracking analysis. The results showed that both CPAF and BaP treatments alone significantly increased MVP release compared to control (Figure 5A,B). Notably, combined treatment with either BaP + CPAF or CPAF + BaP resulted in a further significant increase in MVP release compared to either agent alone. These findings suggest that BaP-induced MVP release may be mediated through the PAFR signaling pathway.

### 3.6. Effect of PAFR and aSMase Inhibition on BaP-Mediated MVP Release

**Figure 6:**
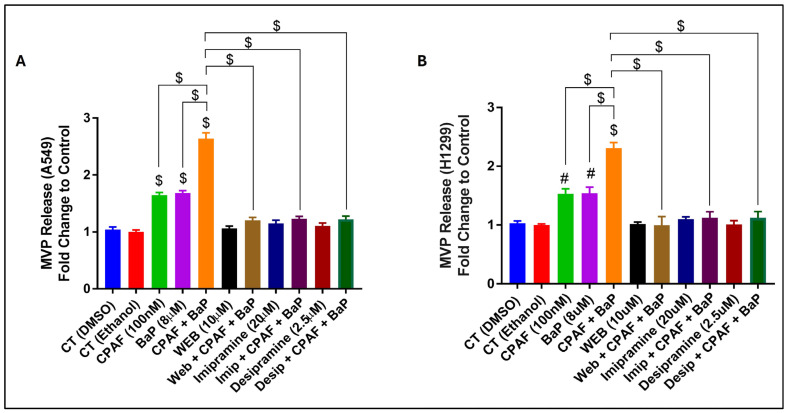
Effects of pharmacological inhibitors of PAFR and aSMase on CPAF ± BaP-induced MVP release in NSCLC: (**A**) A549 and (**B**) H1299 cells were seeded in 6-well plates and treated at 80–90% confluency with BaP (8 µM), CPAF (100 nM), or the CPAF + BaP combination, with or without the PAFR antagonist WEB2086 (10 µM) or the aSMase inhibitors Imipramine (Imip; 20 µM) and Desipramine (Desip; 2.5 µM) for 4 h. The data are expressed as mean ± SEM of at least 3 independent experiments and represented as MVP release fold change to control. The signs # (*p* < 0.001) and $ (*p* < 0.0001) indicate statistically significant difference between the controls and respective treatment groups. The sign $ (*p* < 0.0001) indicates comparison of CPAF + BaP with WEB + CPAF + BaP or Imipramine + CPAF + BaP or Desipramine + CPAF + BaP.

To determine whether BaP-stimulated MVP production occurs through PAFR- and aSMase (an enzyme required for MVP biogenesis)-dependent signaling, cells were treated with BaP and CPAF in the presence or absence of pharmacological inhibitors targeting PAFR and aSMase. Because BaP + CPAF and CPAF + BaP treatments produced comparable levels of MVP release, the CPAF + BaP sequence was selected for subsequent mechanistic studies. The doses of PAFR antagonist WEB2086 and aSMase inhibitor imipramine were taken from our previous published reports [17,18]. However, we did a dose-response effect desipramine that showed reduced cell viability in A549 and H1299 cells in a dose-dependent manner, with 2.5μM identified as non-cytotoxic (Supplementary Figure S1). As shown in Figure 6A,B, BaP and CPAF individually increased MVP release in both the cell lines, whereas combined treatment resulted in a greater magnitude of induction. Pretreatment with the PAFR antagonist WEB2086 reduced CPAF + BaP-induced MVP release to basal levels. A similar suppression was observed when aSMase inhibitors (Imipramine or Desipramine) were used in combination with CPAF + BaP. These findings were similar in both NSCLC cell lines. These effects were consistent across both NSCLC cell lines examined. Collectively, these results indicate that BaP-induced MVP release is mediated through PAFR-dependent signaling and highlight a critical role for the PAFR–aSMase axis in regulating BaP-associated MVP production in lung cancer cells.

## 4. Discussion

The current investigation examined a previously unexplored, PAFR pathway in mediating BaP-induced cellular and molecular responses, including its effects with EGFR-targeted therapies in NSCLC cells. Our findings demonstrate that BaP and CPAF promote cell growth but do not compromise EGFR-TKI antiproliferative efficacy. In addition, BaP induces MVP release, with enhanced effects with CPAF, in a process blocked by pharmacological targeting of PAFR and aSMase pathways.

In this study, exposure of the NSCLC cell lines A549 and H1299 to a wide concentration range of BaP (0.005–16 µM) for up to 72 h did not produce a statistically significant reduction in cell viability. Even at the highest concentration (16 µM), only a modest, non-significant decrease in H1299 cell viability at 48 and 72 h was observed. This result suggests that, under these acute exposure conditions, BaP does not exert any cytotoxic effect on these NSCLC models. This finding is consistent with the previous published studies demonstrating that BaP does not reduce cell viability at low to moderate concentrations in NSCLC models. For example, multiple studies reported that BaP at ≤8 µM did not affect A549 cell viability, although it enhanced migration and invasion [23,24,25]. The absence of a cell survival effect under acute conditions supports the notion that BaP’s pro-tumorigenic role in lung cancer may predominantly involve non-cytotoxic pathways, such as promotion of cell invasion or MVP release. This is consistent with the underlying biology of these models, as KRAS-mutant A549 cells exhibit pro-survival signaling while TP53-null H1299 cells display impaired apoptotic responses, collectively favoring survival [26,27].

Our findings suggested that BaP significantly enhanced the cell migration capacity at both 24 and 48 h time points. When combined with the EGFR-TKIs, Gefitinib and Erlotinib, migration was markedly inhibited, and BaP co-treatment did not significantly negate this inhibition under the experimental conditions tested. Thus, EGFR-TKIs are able to suppress BaP-induced migration in A549 cells. The stimulatory effect of BaP on migration aligns with previous studies by Zhao et al. (2018), which reported that BaP enhanced migration and invasion of NSCLC cells via the TNF-α/NF-κB axis [28]. Moreover, Wu et al. (2020) demonstrated that BaP induced an epithelial–mesenchymal transition (EMT) and migratory behavior in A549 cells via linc00673 in an AHR-dependent manner [23]. The sustained inhibition of BaP-induced cell migration by EGFR-TKIs suggests that, despite oncogenic KRAS signaling in A549 cells, EGFR-dependent pathways continue to contribute to cell motility and remain therapeutically targetable even in the presence of BaP. In addition, it is recognized that wound closure may result from a combination of cellular migration and proliferation. However, given the intrinsic cytotoxicity associated with mitomycin C [29], its use was intentionally avoided to prevent confounding effects that could interfere with the interpretation of BaP- or CPAF-induced responses in the presence or absence of EGFR-TKIs.

Our proliferation experiments showed that both BaP and PAFR agonist CPAF increased proliferation of NSCLC cells. However, when either agent was combined with Gefitinib or Erlotinib, the antiproliferative effects of the EGFR-TKIs remained unchanged, as growth inhibition was equivalent to EGFR-TKI alone. While PAFR signaling has been shown to attenuate the efficacy of cancer therapies [30,31], these findings indicate that BaP exposure or PAFR activation may enhance basal proliferative signaling but does not necessarily mediate resistance to EGFR inhibition in this acute in vitro setting. These findings align with published evidence demonstrating that BaP enhances pro-survival signaling in A549 lung cancer cells. Liu et al. (2023) reported that BaP promotes NSCLC progression by downregulating RNF182, a tumor-suppressive E3 ligase, thereby increasing cell proliferation and survival [32]. In the context of KRAS-mutant and TP53-deficient backgrounds, these findings further support the notion that BaP amplifies tumorigenic signaling without conferring additional resistance to EGFR-targeted therapies.

In the soft-agarose clonogenic assay, both BaP and CPAF markedly increased the number and size of colonies, indicating enhanced anchorage-independent growth and tumorigenic potential. This observation is supported by prior findings showing that BaP promotes malignant transformation in lung models. Wang et al. (2020) demonstrated that BaP exposure increases cancer stem-cell-like properties and tumorigenesis through SOCS3 downregulation, providing mechanistic support for the heightened clonogenicity observed in our study [33]. Similarly, Shahid et al. (2023) reported that BaP drives lung inflammation, toxicity, and carcinogenesis in vivo, confirming its strong tumor-promoting effects [34]. Together, these findings align with our results showing that BaP can enhance the tumorigenic phenotype of NSCLC cells under anchorage-independent conditions. This effect is consistent with the underlying genetic context, as constitutive KRAS signaling and loss of TP53 function are associated with enhanced cell survival, although their functional impact may vary across experimental models.

Despite this enhancement, Gefitinib and Erlotinib retained potent suppression of colony formation across all treatment conditions. Co-treatment with BaP or CPAF did not significantly alter EGFR-TKI responses under the experimental conditions tested, indicating that acute exposure to these agents does not override the EGFR-TKI-mediated cytotoxic response for anchorage-independent growth. This preserved sensitivity suggests that, while BaP and CPAF promote tumorigenic features, they do not significantly compromise EGFR-TKI efficacy in the same context. In contrast to our findings, a study by Jen-Chung et al. found a positive association between Gefitinib treatment and the cytotoxic effects induced by BaP in A549 and H1650 cell lines [7]. The paper indicated that Gefitinib exerts a synergistic effect, enhancing BaP-induced cytotoxic responses. In comparison, our findings did not observe any statistically significant difference in responses when BaP was combined with EGFR-TKIs relative to the effects observed with individual EGFR-TKI treatments. A critical methodological distinction between the two studies may explain this discrepancy. Their study employed a non-toxic concentration of Gefitinib to evaluate its biological effects, whereas our experiments were conducted using IC_50_ and IC_25_ concentrations of Gefitinib to assess cellular responses. Since the outcomes at IC_25_ and IC_50_ concentrations of EGFR-TKIs were consistent and did not demonstrate additional modulation by BaP, subsequent analyses were conducted using the IC_50_ concentration only. This variation in dosing strategy and experimental conditions may have contributed to the differing outcomes reported between the two studies. However, whether chronic or repeated BaP exposure produces different effects, particularly with respect to adaptive resistance mechanisms, DNA damage-associated responses, long-term signaling reprogramming, or the selection of resistant cellular subpopulations, remains unclear and warrants further investigations [35,36].

Our results demonstrate that both BaP and CPAF independently stimulate MVP release in A549 and H1299 cell lines and that co-treatment with BaP and CPAF leads to a greater increase in MVP secretion than either treatment alone. The increased MVP release suggests that BaP and PAFR activation converge on common regulatory pathways, resulting in a coordinated upregulation of MVP formation. These observations are consistent with earlier studies demonstrating that MVP release is tightly regulated by PAFR signaling. Chauhan and colleagues provided direct evidence that targeted therapies, such as Gefitinib and Erlotinib, induce MVP release in NSCLC cells through a PAFR- and aSMase-dependent manner and that pharmacological inhibition of these pathways respectively reduces this MVP release [17]. Our findings extend this mechanistic framework to environmental carcinogen exposure. The observed increase in MVP release across genetically distinct backgrounds following the BaP treatment, especially when combined with CPAF, is consistent with potential involvement of the PAFR–aSMase signaling axis in regulating elevated MVP shedding.

Given the known roles of MVPs in reshaping the tumor microenvironment, promoting immune evasion, and facilitating metastatic progression [37], the pollutant driven enhancement of MVP release observed herein highlights a potential mechanism by which environmental toxicants may contribute to lung cancer aggressiveness. Recent studies have further highlighted the broader pathological significance of extracellular vesicle-mediated signaling networks, including extracellular vesicle–platelet interactions, in NSCLC progression, metastatic dissemination, therapeutic resistance, and liquid biopsy-based biomarker development [38,39,40,41]. Although the present study did not specifically investigate platelet-associated vesicle signaling, these findings further support the relevance of extracellular vesicle-associated pathways in lung cancer biology.

## 5. Conclusions

Overall, this study advances the current understanding of how the environmental pollutant BaP influences cellular behavior of NSCLC, particularly in relation to PAFR-mediated signaling and responses to EGFR-targeted therapies, using a series of in vitro approaches, such as cell viability, migration, proliferation, colony formation, and MVP release. The findings of this paper illustrate a biologically significant intersection between environmental carcinogens and lipid-associated signaling cascades relevant to lung cancer progression. Although BaP did not significantly alter EGFR-TKI-mediated growth inhibition under the experimental conditions tested, its ability to promote migration and enhance MVP biogenesis highlights a potentially important role for environmental toxicants in driving metastatic traits. The parallel effects observed with the pharmacological inhibitors of PAFR and aSMase on BaP and CPAF-induced MVP release are consistent with possible involvement of these signaling pathways; however, further genetic validation is warranted to strengthen these findings. Together, these results provide mechanistic support and a translational foundation for investigating a PAFR blockade as a complementary therapeutic approach to limit pollutant-induced NSCLC progression.

## 6. Limitations and Future Directions

This study was limited to two NSCLC cell lines, A549 and H1299, which provided a controlled experimental framework for evaluating BaP-induced and PAFR-mediated responses in lung cancer under conditions of oxidative stress. Although these cell lines differ in their genetic backgrounds, no differences in the observed outcomes were detected between them. However, these cell lines do not represent classical EGFR-mutant NSCLC models. Therefore, findings related to EGFR-TKI responsiveness should be interpreted within the context of the specific experimental models and conditions used in this study. Future studies should extend these findings to additional genetically diverse NSCLC cell lines, including EGFR-mutant models such as PC9 or HCC827, to determine whether BaP- and CPAF-induced effects represent a generalized phenomenon or are modulated by specific mutational profiles.

Additionally, the present study did not include in vivo models. While in vitro systems are valuable for delineating specific mechanistic pathways, they do not fully recapitulate the complexity of tumor microenvironment interactions, metabolic activation of BaP, or systemic inflammatory influences. To establish physiological relevance, future studies should employ appropriate animal models to evaluate whether BaP exposure modulates EGFR-TKI responsiveness, metastatic potential, MVP dynamics, and PAFR-dependent tumor progression in vivo.

## Data Availability

The original contributions presented in this study are included in the article/Supplementary Materials. Further inquiries can be directed to the corresponding author.

## Supplementary Materials

The following supporting information can be downloaded at: https://www.mdpi.com/article/10.3390/medsci14020301/s1, Figure S1:Effect of Desipramine on (A) A549 and (B) H1299 cell viability.

## Abbreviations

The following abbreviations are used in this manuscript:

NSCLC	Non-small cell lung cancer
EGFR	Epidermal growth factor receptor
TKIs	Tyrosine kinase inhibitors
BaP	Benzo[a]pyrene
BPDE	BaP-7,8-diol-9,10-epoxide
PAFR	Platelet-activating factor-receptor
MVPs	Microvesicle particles
ROS	Reactive oxygen species
HBSS	Hank’s Balanced Salt Solution
DMSO	Dimethyl sulfoxide
SRB	Sulforhodamine B
CPAF	Carbamyl-platelet-activating factor
EMT	Epithelial–mesenchymal transition
