# Supplementary material for: Effects of Benzo[a]pyrene on Targeted Therapy Response and Platelet-Activating Factor-Receptor-Mediated Microvesicle Particle Release in Non-Small Cell Lung Cancer
Source: Med Sci (Basel). 2026 Jun 11;14(2):301. doi: 10.3390/medsci14020301 (PMC13304195; doi:10.3390/medsci14020301)
Supplement: Supplementary file 1 [file medsci-14-00301-s001.zip › medsci-4324097-supplementary.pdf]

6.1 To evaluate the dose-response effects of Desipramine on cell survival.

To determine the suitable concentrations of the aSMase inhibitor, Desipramine, for MVP studies, A549 and H1299 cells were treated with increasing doses of Desipramine for 48 hours, and their effects on cell survival were measured via SRB assay. We observed that Desipramine treatment showed no reduction in cell survival at 2.5 μM and 5 μM doses in both the A549 and H1299 cell lines, and only a moderate decrease in cell survival was observed at 10 μM. Meanwhile, exposure at higher concentrations (20 μM and 30 μM) resulted in a marked loss of cell survival [Figure 1 (A-B)]. These findings demonstrate that 2.5 μM is a non-toxic concentration for Desipramine in A549 and H1299 cell lines, and is therefore appropriate for use in subsequent studies.

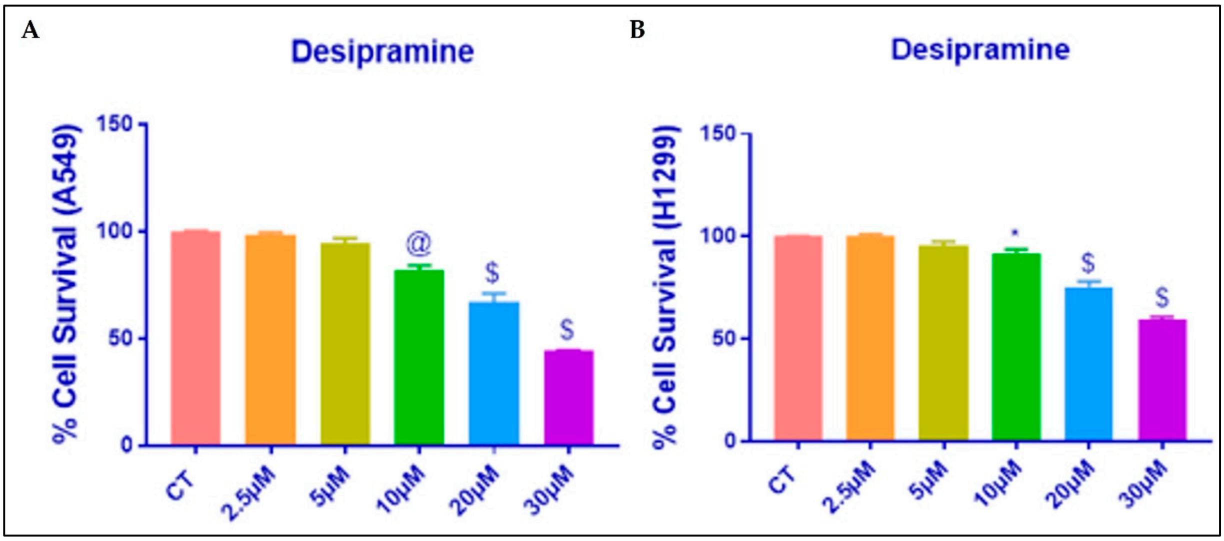

Supplementary Figure S1. **Effect of Desipramine on (A) A549 and (B) H1299 cell viability**. Cells were treated with were treated for 48 hours with increasing concentrations of Desipramine and analyzed using the SRB assay. Data represent mean ± SEM from three independent experiments. Statistical significance denoted as "*" $p<0.05$, "@" $p < 0.01$, "$" $p < 0.0001$ are compared to control.
